# MicroRNAs as Urinary Biomarker for Oncocytoma

**DOI:** 10.1155/2018/6979073

**Published:** 2018-07-16

**Authors:** Melanie von Brandenstein, Monika Schlosser, Jan Herden, Axel Heidenreich, Stefan Störkel, Jochen W. U. Fries

**Affiliations:** ^1^Department of Urology, University Hospital of Cologne, Kerpenerstraße 62, 50931 Cologne, Germany; ^2^Institute of Pathology, Helios Clinic Wuppertal, University Clinic Witten-Herdecke, Heusner St. 40, 42283 Wuppertal, Germany; ^3^Institute of Pathology, University Hospital of Cologne, Kerpenerstraße 62, 50924 Cologne, Germany

## Abstract

The identification of benign renal oncocytoma, its differentiation from malignant renal tumors, and their eosinophilic variants are a continuous challenge, influencing preoperative planning and being an unnecessary stress factor for patients. Regressive changes enhance the diagnostic dilemma, making evaluations by frozen sections or by immunohistology (on biopsies) unreliable. MicroRNAs (miRs) have been proposed as novel biomarkers to differentiate renal tumor subtypes. However, their value as a diagnostic biomarker of oncocytoma in urines based on mechanisms known in oncocytomas has not been exploited. We used urines from patients with renal tumors (oncocytoma, renal cell carcinoma: clear cell, papillary, chromophobe) and with other urogenital lesions. miRs were extracted and detected via qRT-PCR, the respective tumors analyzed by immunohistology. We found isocitrate dehydrogenase 2 upregulated in oncocytoma and oncocytic chromophobe carcinoma, indicating an increased Krebs cycle metabolism. Since we had shown that all renal tumors are stimulated by endothelin-1, we analyzed miRs preidentified by microarray after endothelin-1 stimulation of renal epithelial cells. Four miRs are proposed as *presurgical* urinary biomarkers due to their known regulatory mechanism in oncocytoma: miR-498 (formation of the oncocytoma-specific slice-form of vimentin, Vim3), miR-183 (associated with increased CO_2_ levels), miR-205, and miR-31 (signaling through downregulation of PKC epsilon, shown previously).

## 1. Introduction

The routine histologic diagnosis of a renal oncocytoma is exclusively based on morphologic criteria such as the presence of large epithelial cells in small groups with irregular nuclei and finely granular acidophilic cytoplasm [1]. These, the so-called oncocytes, are considered characteristic showing an excessive amount of mitochondria by electron microscopy [2]. However, the differentiation between the benign oncocytoma [3] and its malignant counterpart, the chromophobe renal cell carcinoma, is a continuous challenge for all diagnostic disciplines, such as radiology, urology, and pathology, since there are no absolute distinctive diagnostic criteria. This differential is further aggravated by the existence of the so-called eosinophilic or oncocytic subtypes of clear cell renal cell carcinoma and chromophobe renal cell carcinoma [4], particularly when secondary regressive tissue alterations such as pseudocystic change and necrosis occur. Even postsurgical morphologic diagnosis relying on immunohistology has no markers allowing a clear-cut distinction between these eosinophilic-appearing tumors [5]. However, this differential is of essential importance for the surgical approach as well as for an adequate postoperative surveillance and ultimately for the prognosis of the patient. Consequently, a presurgical biopsy as well as a frozen section during the surgical operation is unreliable to determine the tumor entity as the decisional basis of the type of operational approach, compromising the chance of nephron-sparing surgery and even requiring unnecessary lymph node resection. To improve presurgical planning, there is an urgent need for noninvasive, specific biomarkers for oncocytoma.

Currently, diagnoses of renal tumors are based on the traditional postresection protein-based analysis by immunohistology [4–7] or by genomic expression analyses to differentiate renal cell carcinoma subgroups [8, 9]. More recently, microRNAs (miRs) have been suggested as a new class of molecules, suitable as biomarkers in tumor *tissue* studies to differentiate renal cell carcinomas [10–17]. miRs are short, noncoding RNAs that can play important roles in cell function and development by targeting mRNA sequences of protein-coding transcripts, resulting either in mRNA cleavage or in repression of productive translation in the cytoplasm. Recently, we have shown that the detection of *urinary* miRs is useful in detecting renal tubular nephrotoxicity [18] as well as renal cell carcinoma [19]. Furthermore, we found that miR-15a, inversely correlated to PKC *α*, is a useful urinary biomarker to differentiate malignant renal tumors with high urinary miR-15a levels from benign renal oncocytoma with low miR-15a levels. However, *upregulated* urinary miRs specifically for benign renal oncocytoma are needed. In this study, we describe four miRs as potential biomarker candidates for *urinary* identification of benign renal oncocytoma.

Previously studied miRs were inducible by stimulation with endothelin- (ET-) 1 [18, 19]. ET-1 is generated by proximal epithelial cells as well as by renal tumors including oncocytoma [19]. It binds via two independent surface receptors, A and B, which are present on the surface of oncocytomas as well as the malignant renal cell carcinomas [19]. ET-1-mediated signal transduction in normal and in tumor renal cells involves protein kinase C isoforms and *β*-catenin [20] and leads to the downregulation of p53 (unpublished data).

Recently, we identified a splice variant of vimentin, vimentin 3 (Vim3), as a specific marker for immunohistologic diagnosis differentiating oncocytoma from malignant renal cell carcinoma [21]. By sequence homology, its unique C-terminal ending after exon 7 has a recognition site for miR-498. These miR and Vim3 are both induced by ET-1 stimulation in normal and tumor cells (unpublished data).

To identify additional miR candidates for this study, we used a previous microarray investigation (miRNA Array 3.0, Affymetrix; 18), showing several other miRs after ET-1 stimulation of renal epithelial cells, such as miR-1826, miR-31, miR-205, and miR-143. These had been described in the literature to downregulate PKC epsilon (in renal cell carcinoma: miR-1826 [22]; in nonrenal cancers such as breast cancer: miR-31 [23]; in prostate cancer: miR-205 [24]; and in the vasculature: miR-143 [25] or p53: miR-205 [26]). Previously, we had shown that PKC epsilon is downregulated in oncocytoma [19] Furthermore, miR-183 has been found upregulated in high CO_2_ levels [27] as occurring in oncocytomas due to dysregulation of the mitochondrial respiratory chain [28], while also being regulated by p53 [29]. Finally, we investigate miR-498, which we observed being specifically expressed in oncocytoma [21].

From the results of this study, we propose four miRs (498, 183, 205, and 31) as specific and highly sensitive candidates for *presurgical urinary* biomarkers to identify benign renal oncocytomas.

miR-183 has been reported to downregulate the enzyme isocitrate dehydrogenase 2 (IDH2) of the Krebs cycle in gliomas [30]. Thus, we studied the expression of IDH2 by immunohistology in different renal tumors, to analyze whether this miR has a similar function in oncocytomas.

## 2. Materials and Methods

Because human materials (renal tissue and urine) were used, procedures were followed as outlined in accordance with ethical standards formulated in the Declaration of Helsinki 1975, with preapproval by the Ethics Committee at the University Hospital, Cologne (reference number 09-232). Informed consent was obtained from all patients to allow the use of samples and clinical data for investigation.

### 2.1. Renal Tumors Tissue Microarray

For tissue microarray analyses, formalin-fixed and paraffinized human renal tissue samples from the archives of the Department of Pathology, University Hospital of Cologne, and from the Department of Pathology, University of Witten-Herdecke, Germany, were used. Tumor and normal tissues were preevaluated for *β*-actin by qPCR to ensure good tissue preservation (see below). From those tissues, core punch biopsy samples (each with a 2 mm diameter) were taken, allowing a simultaneous evaluation of a total of 40 samples per slide [19]. In total, 120 specimens of resected renal cell carcinomas (RCC) and specimens of pericancerous normal renal tissues from the Department of Urology, University Hospital of Cologne, and the Department of Urology, University of Witten-Herdecke, Germany, had been collected. All RCC patients had been treated by radical nephrectomy or partial resection. Of the 110 RCC samples, 10 were papillary RCC. All other groups contained 20 tumor samples (clear cell RCC, eosinophilic/oncocytic variant of clear cell RCC, chromophobe RCC, eosinophilic/oncocytic variant of chromophobe RCC, and oncocytoma) according to the AJCC 2010/UICC 2009 classification. Tumors were staged according to the 2009 revised TNM staging system [31], and clear cell and papillary RCCs were graded according to the Fuhrman four-grade system [32]. Only grade 1 and 2 tumors were used. Clear cell and papillary RCCs were regraded according to ISUP 2016 [33] (clear cell RCC—ISUP grade 1: *n* = 13; grade 2: *n* = 7; eosinophilic variant of clear cell RCC—ISUP grade 1: *n* = 15; grade 2: *n* = 5; and papillary—ISUP grade 1: *n* = 7; grade 2: *n* = 3) No tumor with higher ISUP grade was used.

### 2.2. Preevaluation by Routine Stains

All samples used were routinely analyzed by histological evaluation (light microscopy: hematoxylin-eosin, Periodic-acid Schiff reagent, van Gieson elastic stain, and HALE's colloidal iron stain by three staff pathologists (Cologne: HPD and JWUF; Witten-Herdecke: SS)) independently.

### 2.3. Antibodies

The Vim3 antibody was commercially designed (EZbiolab Inc., Carmel, USA) using the unique C-terminal ending of Vim3 as the target and testes as described before (for detailed information please see patent by the University of Cologne, Brandenstein/Fries, patent number EP 13160876.2-1405) [21].

Renal tumors were preevaluated using the following antibodies: alpha-methylacyl-coenzyme A-racemase (AMACR, p504S; Leica;1 : 50, Wetzler, Germany), anti-carbonic anhydrase IX (ab 1083 51/EPR4151/2; Abcam; 1 : 1000, Cambridge, UK), CD117 (c-kit; clone 12E7; DAKO; 1 : 400, Frankfurt, Germany), cytokeratin 7 (CK7; clone OV-T2 12,730; DAKO; 1 : 6000, Frankfurt, Germany), epithelial membrane antigen (EMA; clone E29; Cell Marque; 1 : 500, Darmstadt, Germany), GATA-3 (clone 650-[823]; Cell Marque; 1 : 1200, Darmstadt, Germany), CD10 (clone 56-C6; Novo Castra; 1 : 20), INI-1 (MRQ-27; Cell Marque; 1 : 100, Darmstadt, Germany), PAX-8 (MRQ-50; Cell Marque; 1 : 100, Darmstadt, Germany), vimentin (V9; clone SP20; Thermo Fisher Scientific; 1 : 800, Frankfurt, Germany), and isocitrate dehydrogenase 2 (IDH2; GTX 628487–100; GeneTex, 1 : 100, Eching, Germany).

### 2.4. Immunohistology of Paraffin-Embedded Tissues

Paraffin-embedded tissue sections (4 *μ*m thick) were deparaffinized by incubation for 2–5 minutes in xylene, followed by 2-3 minutes in 100% ethanol and 1 minute in 95% ethanol, and then rinsed with distilled water. The slides were incubated with a specific serum blocker (anti-rabbit, Frankfurt, Germany) for 30 minutes in order to avoid nonspecific binding. All antibodies are mouse monoclonal origin except CD117 (rabbit polyclonal ab, SP26, Sigma-Aldrich, Frankfurt, Germany). Sections are pretreated with EDTA (Leica Retrieval Solution 2, AR9646, Leica, Wetzler, Germany) except when antibodies against CD10 and vimentin (citrate buffer: Leica Retrieval Solution 1, AR 906, Leica, Wetzler, Germany) or against cytokeratin 7 (Bond Enzyme and Diluent, Leica; manufacturer's instruction) were used. The staining was performed using a BOND MAX stainer (Leica, Göttingen, Germany) and for detection of a polymer with DAB as chromogen as applied. Hemalaun served as a counterstain. Using tissue microarrays (see below) allowed a reevaluation of all samples used in this study by simultaneous immunohistological analysis of 40 tissue samples at once (beyond their preevaluation by routine analysis).

### 2.5. Urine Collection

Urine samples of approximately 50 to 100 mL were selected and stored frozen at −20°C until further use. In tumor patients, urines were obtained immediately prior to operation and 1 week post nephrectomy. The respective tumors were all analyzed by light microscopy and immunohistology as described before for the samples used for tissue microarrays. As samples, a total of 26 renal tumors were analyzed [clear cell RCC (*n* = 10), chromophobe RCC (*n* = 5), papillary RCC (*n* = 6), and benign renal oncocytoma (*n* = 5)]. Furthermore, urines from urothelial carcinoma (*n* = 5), angiomyolipoma (*n* = 2), colon carcinoma (*n* = 5), and from healthy controls (*n* = 5) were collected. Only Fuhrman grade 1/2 tumors were used. Clear cell RCCs with ISUP grade 1 or 2 and papillary RCCs with ISUP grade 1 were selected. Tumors with sarcomatoid differentiation were excluded.

### 2.6. miR Isolation from Urine

For miR isolation from patients' urine, 1 mL of urine was used and added to the Qiazol reagent, mixed, and further used according to the manufacturer's protocol (miRNeasy kit; Qiagen, Hilden, Germany). RNA quantification was accomplished using NanoDrop technology [19].

### 2.7. cDNA Synthesis

The cDNA was obtained from 250 ng of RNA using random primers and SuperScript III reverse transcriptase, according to the manufacturer's protocol (Invitrogen, Darmstadt, Germany). The RT-PCR was performed as previously described [19, 20].

### 2.8. qPCR Data for Tissue Quality Evaluation

From the cDNA, 1 *μ*L was used for quantitative real-time PCR (qPCR) analysis. The experimental settings were as previously described [19, 20]. All experiments were performed in triplicate. Relative fluorescence was calculated using the ΔΔ-CT method, as outlined in user bulletin 2 (PE Applied Biosystems, Darmstadt, Germany). The statistical significance of qPCR values at different time points was assessed by Student's paired *t*-test. 5s rRNA was used as control. For amplification, the following conditions were used: forward primer: GAAUUGCAAGCCACCUGUUG, temp 50°C, 40 cycles. As a reverse primer for 5s rRNA, the universal primer as part of the NCode VILO miRNA cDNA Synthesis Kit (Invitrogen, Darmstadt, Germany) was used. The mirVana qPCR primer set for 5S ribosomal RNA served to normalize results among different samples.  Table 1 provides primer information.

### 2.9. Quantitative Real-Time PCR (qRT-PCR)

The qRT-PCR was performed as previously described [12, 13]. All experiments were performed in triplicate. Relative fluorescence was calculated using the ΔΔ-CT method, as outlined in User Bulletin 2 (PE Applied Biosystems, Darmstadt, Germany). The statistical significance of qPCR values at different time points was assessed by Student's paired *t*-test.

### 2.10. Statistical Analysis

For statistical analysis, the GraphPrism 5 (San Diego, California, USA) program was used. Analysis of variance (ANOVA) was performed, and the significant differences were calculated and indicated by stars (^∗^*p* < 0.05, ^∗∗^*p* < 0.01, and ^∗∗∗^*p* < 0.001). All differences without indication were not statistically significant.

## 3. Results

### 3.1. Presurgical Urinary miR Analysis

**Figure 1:**
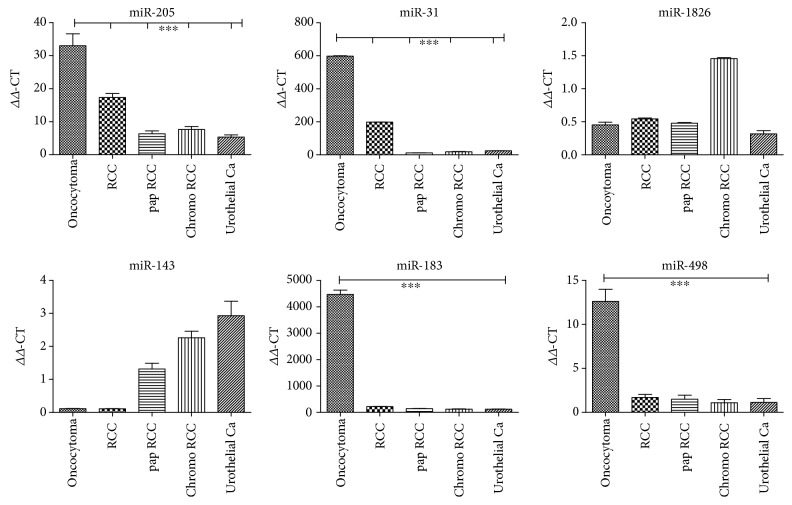
Presurgical urinary levels of miRs for renal tumor diagnostic. miR-205, miR-31, miR-498, and miR-183 are highly expressed in the urine of oncocytoma patients (^∗∗∗^*p* < 0.001), while miR-1826 and miR-143 are unsuitable as oncocytoma marker. Oncocytoma; RCC: clear cell renal cell carcinoma; pap RCC: papillary renal cell carcinoma; chromo RCC: chromophobe renal cell carcinoma; urothelial Ca: urothelial carcinoma.

The levels of six miRs in the urines of renal and urological tumors were analyzed by PCR (Figure 1**)**. *miR-205* levels in oncocytomas were about twice as high as those in renal cell carcinomas and even higher compared to other malignant tumors, reaching a high statistical significance. Results from the investigation of *miR-31* yielded high positive values for oncocytomas, being the second highest for all oncocytoma-miRs selected. All other renal tumors and particularly the urothelial carcinoma were much lower with a high statistical significance. *miR-1826* had the lowest of all levels in the urine and was foremost expressed in chromophobe carcinoma with no statistical significance. *miR-143* showed low urinary values for oncocytoma and was not significantly upregulated for any of the malignant renal tumors. In contrast, the highest levels were obtained in the urine of urothelial cancer patients. *miR-183* demonstrates the highest level of all miRs investigated for oncocytoma with a high statistical significance. In addition, urines from angiomyolipoma and colon carcinoma were analyzed with barely detectable values for this miR (data not shown).

Since *miR-498* is associated with the formation vimentin 3, it was investigated with particular emphasis on oncocytoma. Extraction and PCR analysis revealed a statistically significant upregulation in oncocytomas versus all other renal cell carcinoma entities.

### 3.2. Postsurgical Urinary miR Analysis

**Figure 2:**
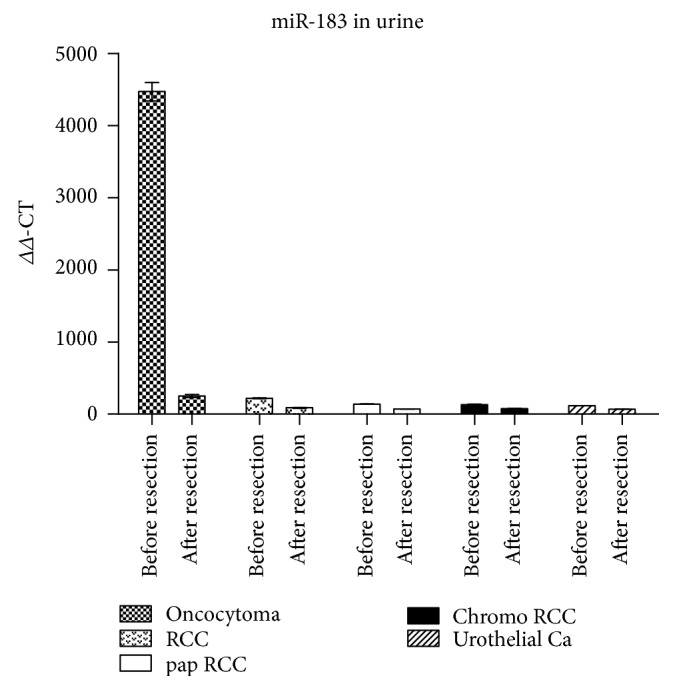
Levels of miR-183 in the urine of renal tumor patients after tumor resection. Residual miRs in urine detectable, being highly reduced in oncocytoma. Oncocytoma; RCC: clear cell renal cell carcinoma; pap RCC: papillary renal cell carcinoma; chromo RCC: chromophobe renal cell carcinoma; urothelial Ca: urothelial carcinoma.

Since miR-183 appears to be our best candidate in the urinary diagnostic approach to identify oncocytomas, we study its level in patients' urines after about 1 week after surgery before being released from the hospital. Its level drops to about 1/20th of pretumor values while the oncocytoma was still present, while values in other renal tumors are basically unchanged (Figure 2).

As miR is related to Vim3, miR-498 was also analyzed postsurgically. As expected, no miR was detectable in urines from oncocytoma or renal cell carcinoma patients (data not shown).

### 3.3. Isocitrate Dehydrogenase Immunohistology

**Figure 3:**
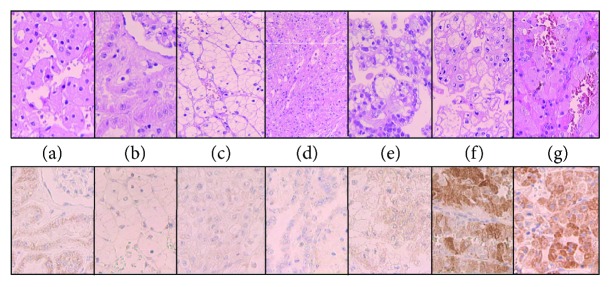
Immunohistology of isocitrate dehydrogenase (IDH2) expression in different renal tumors. IDH2 is expressed in eosinophilic chromophobe renal cell carcinoma and oncocytoma, not in other renal carcinomas. (a) Normal kidney, (b) RCC: clear cell renal cell carcinoma, (c) eosinophilic RCC, (d) papillary RCC, (e) chromophobe RCC, (f) eosinophilic chromophobe RCC, and (g) oncocytoma.

Renal cell tumors and their eosinophilic/oncocytic variants were analyzed by immunohistology for isocitrate dehydrogenase 2 (IDH2) expression using tissue microarrays. IDH2 was strongly positive in oncocytoma (18/20) and in the eosinophilic/oncocytic variant of chromophobe renal cell carcinoma (5/20). All other renal tumors, including the oncocytic variant of clear cell carcinoma, are negative (Figure 3). The normal kidney as control shows slight staining in proximal and slight to regionally moderate staining in the distal tubular epithelium.

## 4. Discussion

This study presents data that miR-498, miR-183, miR-205, and miR-31 are suitable *urinary* biomarkers for the *presurgical* diagnosis of oncocytoma. In our opinion, their suitability is based on the following aspects: their significant levels of detection in comparison to that of malignant renal tumors and other urological lesions, a marked decrease of their levels after tumor resection, and their function in signal transduction as previously shown by us, or already known from the literature. From our previous experience [18, 19], it is helpful to identify the potential regulatory pathways in which the selected miR candidate plays an identified role, not only to understand its importance in the signaling cascade and thereby better evaluate the degree of its specificity as a biomarker for a given tumor but also to provide potential targets for future therapeutic interventions [19].

Our investigation originated from the previously published observation that Vim3 is a protein in the cytoplasm of oncocytomas [21], which allows specific differentiation from other urological/renal tumors. We showed that Vim3 is a splice form of full-length vimentin, which has a 3′ binding site for miR-498, leading to induction of VIM3 by miR-498 after ET-1 stimulation (data not shown). Thus, in analogy to the urinary detectability of miR-15a in renal cell cancer, which was selected based on an identified signaling mechanism [19], we postulated that miR-498 could also be used as a urinary biomarker to identify oncocytomas via urine analysis. The level of detectability in the urine of oncocytomas versus renal cell carcinoma as well as controls strongly supports this proposal [Figure 1].

Looking into further significant mechanism specifically identified in oncocytomas, we found reports in the literature describing miR-183 levels being increased by high levels of CO_2_ [27]. This was attributed in renal oncocytomas due to their unique mitochondrial dysfunction caused by the absence of the mitochondrial complex I [34]. The mitochondrial DNA content as well as the amount and the catalytic activity of several oxidative phosphorylation (OXPHOS) complexes has been shown to be increased in oncocytoma as a potential compensatory mechanism [28]. This dysregulation causes an increase in CO_2_, which in a chemical reaction is degraded in H^+^ ions and hydrocarbonate [35] by an increased amount of carboanhydrase found in oncocytomas [36]. In parallel to the observation that increased tissue levels of miR-15a also led to a high level of this miR in the urine in malignant renal cell carcinomas, we investigated the role of miR-183 as a potential urinary biomarker for oncocytomas. This resulted in the highest amounts of all miRs analyzed in onocytoma urines (Figure 1).

While increased levels of miR-183 have been described in the tissue of other nonrenal cancers such as bladder [37] and colon cancer [38] and in individual renal carcinoma cell lines [39], our urine analysis from different types of RCCs and bladder cancer patients could not detect any significant amounts only remotely close to the ones in urines from oncocytoma patients. Thus, we conclude that miR-183 is a unique and independent marker for renal oncocytomas, which are the only urological tumors known so far with the necessary mitochondrial dysregulation to induce this miR.

In this context, it is noteworthy that in human alveolar carcinoma cells, increased amounts of CO_2_ are able to downregulate the enzyme isocitrate dehydrogenase 2 (IDH2) of the Krebs cycle [27]. Tanaka et al. [30] found that high expression levels of miR-183 in various types of gliomas are also associated with a downregulation of IDH2, which has complementary sequences to miR-183 in its 3′-untranslated region. In contrast to expectations based on these studies, we observed a strong IDH2 expression by immunohistology in oncocytomas and to a lesser degree in eosinophilic/oncocytic variants of chromophobe carcinoma by TMA (Figure 3). Interestingly, IDH2 could not be identified in all 10 cases of eosinophilic variants of clear cell renal cell carcinomas (Figure 3). Thus, oncocytic differentiation in chromophobe RCC could be interpreted as a remaining feature from an oncocytic tumor which can be transformed into a chromophobe RCC by acquiring p53 mutations [40]. This interpretation is based on the observation in the literature of the existence of a so-called type II oncocytoma with potential malignant behavior. It is characterized morphologically by enlarged oncocytes measuring 50 *μ*m in diameter [40]. Mutations in the tumor suppressor gene p53 are proposed to be the reason for changes in the gene expression profile of the benign oncocytoma [40] leading to a progression into an eosinophilic/oncocytic subtype of chromophobe RCC [41]. Otherwise, there is a high degree of homology in the gene expression profile between a benign oncocytoma and a chromophobe RCC [42]. Since these insights are recently published, for example, in 2014/2015, a study describing the frequency of benign oncocytomas developing into these type II tumors and subsequently into a carcinoma is yet not available.

miR-205 has been evaluated as a potential biomarker in a variety of different adenocarcinomas, being either downregulated (stomach [43], colon [44], breast [45], and endocrine system [46]) or upregulated (cervix, [47], endometrial carcinoma, [48], ovary, [49], nasopharyngeal carcinoma [50], small cell lung cancers [51], aggressive mucinous adenocarcinoma of the colon [52], and pancreas [53]). Most noteworthy for our purpose of specificity as an oncocytoma marker, miR-205 is downregulated in prostate cancers [54]. Furthermore, most publications (except [55, 56]) also report a downregulation in bladder cancer, including a review of 108 relevant publications [57, 58]. In addition, a study focusing on miR levels in biofluids (serum, urine) has found that miR-205 was so low that it can no longer be considered a useful biomarker [59]. In our study in 5 urines from urothelial carcinoma, we were unable to detect increased levels which might interfere with those detected in the urine of patients with oncocytoma (see Figure 1).

Finally, we observed that miR-31 is highly expressed in urines from an oncocytoma tumor patient (Figure 1). Besides downregulating PKC epsilon [19], no other analysis has been reported with this miR in oncocytoma. In contrast, it is frequently altered in a large variety of cancers. The functional role of miR-31 is extremely complex: both tumor suppressive and oncogenic roles in different tumor types have been described [60]. In a large meta-analysis including a total of 14 studies with 1397 cancer patients and 1039 controls, miR-31 was identified as a circulatory miR in serum in carcinomas of the lung, the colon, the pancreas, the breast, and the oral cavity [61]. However, in urological tumors, miR-31 is reduced as shown in Figure 1, which is in agreement with studies in the literature (bladder cancer [62]; prostate cancer [63]). Thus, the use of miR-31 as a urinary marker for oncocytoma is not compromised by secretion into the urine from other urological tumors.

In principle, there are several different ways of analyzing miRs for diagnosing renal tumors. Besides resection specimens, a new approach called liquid biopsy is available using either serum or urine. While serum diagnostics of mutated genes have been useful in some carcinomas, the studies applying liquid biopsy technology to renal tumors yielded disappointing results. Corrò et al. [64] analyzed clear cell RCCs with VHL genetic mutations by next-generation sequencing (NGS) and Taqman mutation assay. While they could detect KRAS-mutated colon carcinoma, all renal tumors were negative by NGS, and only 1 out of 9 samples was positive by mutation assay. Using miRs extracted from the serum, our previous study [19] could principally identify miR-15a in very low quantities but these were unrelated to the presence or absence of renal cell carcinomas. Similarly, Wulfken et al. [65] reported that miR-1233 is detectable in serum, but its reported upregulation in RCCs as well as in oncocytomas and angiomyolipomas is not useful for benign versus malignant tumor differentiation. Several other studies reported miRs upregulated in renal cancer [29, 66–69]. However, although all studies used serum from clear cell renal carcinomas, there was not a single miR found in common, which may be based on yet unidentified subgroups of genetically different clear cell renal carcinomas not recognizable by morphology alone [70]. Here, a larger study comparing these results and potentially elucidating the differences in identifying miRs in the serum as well as the technical limitations of such an assay is urgently needed. In addition, no study involving serum miRs to specifically diagnose oncocytoma has yet been reported.

However, for urological diagnosis of renal tumors, the least invasive route would be the analysis of urine samples, being easily replicable, with minimal contaminating cellular elements, and lending itself for eventual self-detection or surveillance by the patient, when an adequate test system becomes available. Besides the studies from our group using urine for the detection of RCCs by high levels of miR-15a [19], there are only two other reports in renal cancer using the urine as material for a liquid biopsy. Fedorko et al. [71] detected let-7 miRs in urine supernatant as a potential diagnostic approach in nonmetastatic clear cell renal cell carcinoma. A second study by Butz et al. [72] screened urinary miRs for differential diagnosis between patients with renal tumors (malignant and benign) and controls. While the main focus was the differentiation of renal cell carcinomas from healthy controls, they claimed to have detected oncocytomas with a 75% sensitivity and an 87% specificity using receiver operating characteristic analysis (ROC; [73]). These values seem insufficient to differentiate individual cases of oncocytoma in a daily setting. Regrettably, their actual PCR values are not available for comparison. In addition, these results needed the combined calculated ROC analysis of two miRs, miR-126-3p and miR-486-5p. No postoperative values were determined. No analysis of any other urological lesions was mentioned. Neither of the chosen miRs has been ever reported in RCCs or oncocytoma: miR-486 is found in several different carcinomas (non-small cell lung cancer, hepatocellular carcinoma, esophageal cancer, ovarian cancer, breast cancer, and others), while miR-126-3p is predominantly known in the literature for playing a role in endothelial cell regulation. The authors do not mention any pathway or mechanism for either miRs known to be specifically relevant for oncocytoma.

The currently most significant limitation of a miR-based analysis is the lack of a sensitive and user-friendly urine test system for monitoring any urological tumor. The presently available methods are relatively complex, needing expertise and a special equipment most likely available in a larger clinical than a private practice setting. However, miRs appear to be ideal future biomarkers for urinary analysis because of their presence and stability in urine [19], their relatively easy detectability (by qRT-PCR), their stability even after freeze-thaw cycles, and their specificity to tissue or disease states. Therefore, it is noteworthy that Smith et al. [74] reported a promising approach to detect urinary miRs with high sensitivity via sulfonamide-bound antisense hybridisation. The authors claim that this method has several significant advantages over circulating biomarker analysis including safety, cost, speed, and ease of conversion. While comprehensive studies evaluating this new approach are needed, it seems likely that urinary miRs as a diagnostic tool as presented in our study for oncocytoma may be used in routine diagnostics sooner than expected.

Finally, while a study like this provides a new promising approach for future diagnostics, for clinical applicability, a double-blinded study using a much larger number of urines from tumor and control patients is needed. Not only will this provide the necessary proof of validity but also it will hopefully demonstrate the robustness and ease of this detection method. Furthermore, potential limitations regarding urine amount and contaminants (blood, bacteria, and necrotic cells) can be assessed, and rare new renal tumor entities can be included for differential diagnosis [75].

In summary, we present four miRs (miR-498, miR-183, miR-205, and miR-31) as specific *urinary* biomarkers for the *presurgical* diagnosis of the classic benign, renal oncocytoma, of which miR-498 and miR183 are selected based on specific metabolic/structural features not found in other renal/urologic tumors.

## Figures and Tables

**Table 1:** Primer sequence.

Primer sequence	
183: >hsa-miR-183-5p	UAUGGCACUGGUAGAAUUCAC
31: >hsa-miR-31-5p	AGGCAAGAUGCUGGCAUAGCU
143: >hsa-miR-143-5p	GGUGCAGUGCUGCAUCUCUGG
205: >hsa-miR-205-5p	UCCUUCAUUCCACCGGAGUCU
1826: >hsa-miR-1826	AUUGAUCAUCGACACUUCGAA
498: >hsa-miR-498	UUUCAAGCCAGGGGGCGUUUUU
5s rRNA	GAAUUGCAAGCCACCUGUUG

## Data Availability

The statement about where data supporting the results reported in a published article can be found (see References). *Hyperlinks* to publicly archived datasets analyzed or generated during the study are not applicable.

## References

[1] Hamperl H. (1936). Über das vorkommen von onkocyten in verschiedenen organen und ihren geschwülsten. *Virchows Archiv für Pathologische Anatomie und Physiologie und für Klinische Medizin*.

[2] Kataoka R., Hyo Y., Hoshiya T., Miyahara H., Matsunaga T. (1991). Ultrastructural study of mitochondria in oncocytes. *Ultrastructural Pathology*.

[3] Klein M. J., Valensi Q. J. (1976). Proximal tubular adenomas of kidney with so-called oncocytic features. A clinicopathologic study of 13 cases of a rarely reported neoplasm. *Cancer*.

[4] Kuroda N., Tanaka A., Yamaguchi T. (2013). Chromophobe renal cell carcinoma, oncocytic variant: a proposal of a new variant giving a critical diagnostic pitfall in diagnosing renal oncocytic tumors. *Medical Molecular Morphology*.

[5] Fernández-Aceñero M. J., Cazorla A., Manzarbeitia F. (2011). Immunohistochemistry for the differential diagnosis of renal tumors with oncocytic features. *Urologic Oncology*.

[6] Walter B., Hartmann A., Hofstädter F. (2012). Immunohistochemical marker panel differentiates between the three most common subtypes of renal cell carcinoma independent from histomorphologic criteria. *Virchows Archiv*.

[7] Bing Z., Lal P., Lu S., Ziober A., Tomaszewski J. E. (2013). Role of carbonic anhydrase IX, *α*-methylacyl coenzyme a racemase, cytokeratin 7, and galectin-3 in the evaluation of renal neoplasms: a tissue microarray immunohistochemical study. *Annals of Diagnostic Pathology*.

[8] Beleut M., Zimmermann P., Baudis M. (2012). Integrative genome-wide expression profiling identifies three distinct molecular subgroups of renal cell carcinoma with different patient outcome. *BMC Cancer*.

[9] Tan P. H., Cheng L., Rioux-Leclercq N. (2013). Renal tumors: diagnostic and prognostic biomarkers. *The American Journal of Surgical Pathology*.

[10] Jung M., Mollenkopf H. J., Grimm C. (2009). MicroRNA profiling of clear cell renal cell cancer identifies a robust signature to define renal malignancy. *Journal of Cellular and Molecular Medicine*.

[11] Juan D., Alexe G., Antes T. (2010). Identification of a microRNA panel for clear-cell kidney cancer. *Urology*.

[12] Fridman E., Dotan Z., Barshack I. (2010). Accurate molecular classification of renal tumors using microRNA expression. *The Journal of Molecular Diagnostics*.

[13] Redova M., Svoboda M., Slaby O. (2011). MicroRNAs and their target gene networks in renal cell carcinoma. *Biochemical and Biophysical Research Communications*.

[14] White N. M. A., Bao T. T., Grigull J. (2011). miRNA profiling for clear cell renal cell carcinoma: biomarker discovery and identification of potential controls and consequences of miRNA dysregulation. *The Journal of Urology*.

[15] Youssef Y. M., White N. M. A., Grigull J. (2011). Accurate molecular classification of kidney cancer subtypes using microRNA signature. *European Urology*.

[16] Silva-Santos R. M., Costa-Pinheiro P., Luis A. (2013). MicroRNA profile: a promising ancillary tool for accurate renal cell tumour diagnosis. *British Journal of Cancer*.

[17] Ge Y.-Z., Xin H., Lu T.-Z. (2015). MicroRNA expression profiles predict clinical phenotypes and prognosis in chromophobe renal cell carcinoma. *Scientific Reports*.

[18] Loeser H., von Brandenstein M., Herschung A., Schlosser M., Büttner R., Fries J. W. U. (2015). ET-1 induced downregulation of MRP2 via miRNA 133a - a marker for tubular nephrotoxicity?. *American Journal of Nephrology*.

[19] von Brandenstein M., Pandarakalam J. J., Kroon L. (2012). MicroRNA 15a, inversely correlated to PKC*α*, is a potential marker to differentiate between benign and malignant renal tumors in biopsy and urine samples. *The American Journal of Pathology*.

[20] Gerstung M., Roth T., Dienes H. P., Licht C., Fries J. W. U. (2007). Endothelin-1 induces NF-*κ*B via two independent pathways in human renal tubular epithelial cells. *American Journal of Nephrology*.

[21] von Brandenstein M., Puetz K., Schlosser M. (2015). Vimentin 3, the new hope, differentiating RCC versus oncocytoma. *Disease Markers*.

[22] Hirata H., Hinoda Y., Ueno K., Nakajima K., Ishii N., Dahiya R. (2012). MicroRNA-1826 directly targets beta-catenin (CTNNB1) and MEK1 (MAP2K1) in VHL-inactivated renal cancer. *Carcinogenesis*.

[23] Körner C., Keklikoglou I., Bender C., Wörner A., Münstermann E., Wiemann S. (2013). MicroRNA-31 sensitizes human breast cells to apoptosis by direct targeting of protein kinase C ϵ (PKCϵ). *The Journal of Biological Chemistry*.

[24] Gandellini P., Folini M., Longoni N. (2009). miR-205 exerts tumor-suppressive functions in human prostate through down-regulation of protein kinase C. *Cancer Research*.

[25] Quintavalle M., Elia L., Condorelli G., Courtneidge S. A. (2010). MicroRNA control of podosome formation in vascular smooth muscle cells in vivo and in vitro. *The Journal of Cell Biology*.

[26] Chang C. J., Chao C. H., Xia W. (2011). p53 regulates epithelial-mesenchymal transition and stem cell properties through modulating miRNAs. *Nature Cell Biology*.

[27] Vohwinkel C. U., Lecuona E., Sun H. (2011). Elevated CO(2) levels cause mitochondrial dysfunction and impair cell proliferation. *The Journal of Biological Chemistry*.

[28] Simonnet H., Demont J., Pfeiffer K. (2003). Mitochondrial complex is deficient in renal oncocytomas. *Carcinogenesis*.

[29] Cheng T., Wang L., Li Y., Huang C., Zeng L., Yang J. (2013). Differential microRNA expression in renal cell carcinoma. *Oncology Letters*.

[30] Tanaka H., Sasayama T., Tanaka K. (2013). MicroRNA-183 upregulates HIF-1*α* by targeting isocitrate dehydrogenase 2 (IDH2) in glioma cells. *Journal of Neuro-Oncology*.

[31] Novara G., Ficarra V., Antonelli A. (2010). Validation of the 2009 TNM version in a large multi-institutional cohort of patients treated for renal cell carcinoma: are further improvements needed?. *European Urology*.

[32] Minervini A., Lilas L., Minervini R., Selli C. (2002). Prognostic value of nuclear grading in patients with intracapsular (pT1-pT2) renal cell carcinoma. Long-term analysis in 213 patients. *Cancer*.

[33] Wittekind C., Moch H. (2016). WHO-ISUP-graduierungssystem für nierenkarzinome. *Der Pathologe*.

[34] Zimmermann F. A., Mayr J. A., Feichtinger R. (2011). Respiratory chain complex I is a mitochondrial tumor suppressor of oncocytic tumors. *Frontiers in Bioscience*.

[35] Zheng Y.-J., Merz K. M. (1992). Mechanism of the human carbonic anhydrase II-catalyzed hydration of carbon dioxide. *Journal of the American Chemical Society*.

[36] Kuwahara H., Shimazaki M., Kadoya Y. (1989). Immunohistochemical localization of two types of carbonic anhydrase isozymes in oncocytoma and oncocytic epithelial cells. *Osaka City Medical Journal*.

[37] Yamada Y., Enokida H., Kojima S. (2011). MiR-96 and miR-183 detection in urine serve as potential tumor markers of urothelial carcinoma: correlation with stage and grade, and comparison with urinary cytology. *Cancer Science*.

[38] Sarver A. L., Li L., Subramanian S. (2010). MicroRNA miR-183 functions as an oncogene by targeting the transcription factor EGR1 and promoting tumor cell migration. *Cancer Research*.

[39] Qiu M., Liu L., Chen L. (2014). MicroRNA-183 plays as oncogenes by increasing cell proliferation, migration and invasion via targeting protein phosphatase 2A in renal cancer cells. *Biochemical and Biophysical Research Communications*.

[40] Joshi S., Tolkunov D., Aviv H. (2015). The genomic landscape of renal oncocytoma identifies a metabolic barrier to tumorigenesis. *Cell Reports*.

[41] Guo J. Y., Karsli-Uzunbas G., Mathew R. (2013). Autophagy suppresses progression of K-ras-induced lung tumors to oncocytomas and maintains lipid homeostasis. *Genes & Development*.

[42] Strohecker A. M., White E. (2014). Targeting mitochondrial metabolism by inhibiting autophagy in BRAF-driven cancers. *Cancer Discovery*.

[43] Xu C., Li M.’e., Zhang L. (2016). MicroRNA-205 suppresses the invasion and epithelial-mesenchymal transition of human gastric cancer cells. *Molecular Medicine Reports*.

[44] Yang X., Yang L., Ma Y., Zhao X., Wang H. (2017). MicroRNA-205 mediates proteinase-activated receptor 2 (PAR2) -promoted cancer cell migration. *Cancer Investigation*.

[45] Adhami M., Haghdoost A. A., Sadeghi B., Afshar R. M. (2018). Candidate miRNAs in human breast cancer biomarkers: a systematic review. *Breast Cancer*.

[46] Lima C. R., Gomes C. C., Santos M. F. (2017). Role of microRNAs in endocrine cancer metastasis. *Molecular and Cellular Endocrinology*.

[47] Pang H., Yue X. (2017). MiR-205 serves as a prognostic factor and suppresses proliferation and invasion by targeting insulin-like growth factor receptor 1 in human cervical cancer. *Tumour Biology*.

[48] Chung T. K. H., Cheung T. H., Huen N. Y. (2009). Dysregulated microRNAs and their predicted targets associated with endometrioid endometrial adenocarcinoma in Hong Kong women. *International Journal of Cancer*.

[49] Li J., Hu K., Gong G. (2017). Upregulation of MiR-205 transcriptionally suppresses SMAD4 and PTEN and contributes to human ovarian cancer progression. *Scientific Reports*.

[50] Mao Y., Wu S., Zhao R., Deng Q. (2016). MiR-205 promotes proliferation, migration and invasion of nasopharyngeal carcinoma cells by activation of AKT signalling. *The Journal of International Medical Research*.

[51] Duan B., Guo T., Sun H., Cai R., Rui Q., Xi Z. (2017). miR-205 as a biological marker in non-small cell lung cancer. *Biomedicine & Pharmacotherapy*.

[52] Eyking A., Reis H., Frank M., Gerken G., Schmid K. W., Cario E. (2016). MiR-205 and MiR-373 are associated with aggressive human mucinous colorectal Cancer. *PLoS One*.

[53] Ali S., Dubaybo H., Brand R. E., Sarkar F. H. (2015). Differential expression of microRNAs in tissues and plasma co-exists as a biomarker for pancreatic cancer. *Journal of Cancer Science & Therapy*.

[54] Yamada Y., Nishikawa R., Kato M. (2018). Regulation of HMGB3 by antitumor miR-205-5p inhibits cancer cell aggressiveness and is involved in prostate cancer pathogenesis. *Human Genetics*.

[55] Ratert N., Meyer H. A., Jung M. (2013). miRNA profiling identifies candidate mirnas for bladder cancer diagnosis and clinical outcome. *The Journal of Molecular Diagnostics*.

[56] Fang Z., Dai W., Wang X. (2016). Circulating miR-205: a promising biomarker for the detection and prognosis evaluation of bladder cancer. *Tumour Biology*.

[57] Ganji S. M., Saidijam M., Amini R. (2017). Evaluation of microRNA-99a and microRNA-205 expression levels in bladder cancer. *International Journal of Molecular Medicine*.

[58] Mitash N., Tiwari S., Agnihotri S., Mandhani A. (2017). Bladder cancer: micro RNAs as biomolecules for prognostication and surveillance. *Indian Journal of Urology*.

[59] Armstrong D. A., Green B. B., Seigne J. D., Schned A. R., Marsit C. J. (2015). MicroRNA molecular profiling from matched tumor and bio-fluids in bladder cancer. *Molecular Cancer*.

[60] Laurila E. M., Kallioniemi A. (2013). The diverse role of miR-31 in regulating cancer associated phenotypes. *Genes, Chromosomes & Cancer*.

[61] Ma Y., Chen Y., Lin J. (2017). Circulating miR-31 as an effective biomarker for detection and prognosis of human cancer: a meta-analysis. *Oncotarget*.

[62] Wang S., Li Q., Wang K. (2013). Decreased expression of microRNA-31 associates with aggressive tumor progression and poor prognosis in patients with bladder cancer. *Clinical & Translational Oncology*.

[63] Fuse M., Kojima S., Enokida H. (2012). Tumor suppressive microRNAs (miR-222 and miR-31) regulate molecular pathways based on microRNA expression signature in prostate cancer. *Journal of Human Genetics*.

[64] Corrò C., Hejhal T., Poyet C. (2017). Detecting circulating tumor DNA in renal cancer: an open challenge. *Experimental and Molecular Pathology*.

[65] Wulfken L. M., Moritz R., Ohlmann C. (2011). MicroRNAs in renal cell carcinoma: diagnostic implications of serum miR-1233 levels. *PLoS One*.

[66] Redova M., Poprach A., Nekvindova J. (2012). Circulating miR-378 and miR-451 in serum are potential biomarkers for renal cell carcinoma. *Journal of Translational Medicine*.

[67] Teixeira A. L., Ferreira M., Silva J. (2014). Higher circulating expression levels of miR-221 associated with poor overall survival in renal cell carcinoma patients. *Tumour Biology*.

[68] Zhao A., Li G., Péoc'h M., Genin C., Gigante M. (2013). Serum miR-210 as a novel biomarker for molecular diagnosis of clear cell renal cell carcinoma. *Experimental and Molecular Pathology*.

[69] Zhai Q., Zhou L., Zhao C. (2012). Identification of miR-508-3p and miR-509-3p that are associated with cell invasion and migration and involved in the apoptosis of renal cell carcinoma. *Biochemical and Biophysical Research Communications*.

[70] Brugarolas J. (2014). Molecular genetics of clear-cell renal cell carcinoma. *Journal of Clinical Oncology*.

[71] Fedorko M., Juracek J., Stanik M. (2017). Detection of let-7 miRNAs in urine supernatant as potential diagnostic approach in non-metastatic clear-cell renal cell carcinoma. *Biochemia Medica*.

[72] Butz H., Nofech-Mozes R., Ding Q. (2016). Exosomal microRNAs are diagnostic biomarkers and can mediate cell-cell communication in renal cell carcinoma. *European Urology Focus*.

[73] Song X., Zhou X. H. (2005). A marginal model approach for analysis of multi-reader multi-test receiver operating characteristic (ROC) data. *Biostatistics*.

[74] Smith D. A., Newbury L. J., Drago G., Bowen T., Redman J. E. (2017). Electrochemical detection of urinary microRNAs via sulfonamide-bound antisense hybridisation. *Sensors and Actuators B: Chemical*.

[75] Srigley J. R., Delahunt B., Eble J. N. (2013). The International Society of Urological Pathology (ISUP) Vancouver classification of renal neoplasia. *The American Journal of Surgical Pathology*.

